# Diverting Loop Ileostomy as an Alternative to Emergent Colectomy in Acute Severe Ulcerative Colitis Flare following Checkpoint-Inhibitor Therapy

**DOI:** 10.1093/ibd/izaf187

**Published:** 2025-09-19

**Authors:** Ethan X Tan, Vinna An, Mayur Garg

**Affiliations:** Department of Gastroenterology, Northern Health, Epping, Victoria, Australia; Department of Colorectal Surgery, Eastern Health, Box Hill, Victoria, Australia; Department of Gastroenterology, Northern Health, Epping, Victoria, Australia; Department of Medicine, University of Melbourne, Parkville, Victoria, Australia

**Keywords:** Ulcerative colitis, Checkpoint inhibitor, Ileostomy, Melanoma

Checkpoint inhibitors are increasingly prescribed for malignancies and may worsen preexisting UC in up to 40% of patients.^1^ Most patients with severe disease require corticosteroids, one-third require checkpoint-inhibitor discontinuation and escalation to biologic therapy, and up to 5% require surgery.^1^ We describe the first case of successful loop ileostomy and reversal in checkpoint-inhibitor worsening of UC.

An 81-year-old man with previously well-controlled ulcerative proctitis diagnosed in 1970, with documented symptomatic, endoscopic, and histologic remission on sulfasalazine, was referred in 2022 with increased blood-stained diarrhea up to 5 bowel movements per day for the previous week. He was diagnosed with metastatic melanoma 4 months earlier and commenced taking nivolumab 3 months earlier and ipilimumab 6 weeks earlier. Despite withholding immunotherapy, maximizing oral and topical 5-aminosalicylate (5-ASA) therapy, and commencement of oral steroids, he progressed to severe left-sided colitis over the following 6 weeks, with >10 blood-stained bowel actions per day, a Mayo endoscopic subscore of 3, with C-reactive protein (CRP) >100 mg/L, and albumin <20 g/L. He also had severe inflammatory peripheral arthritis. Stool cultures were negative, including for *Clostrioides difficile*. Cytomegalovirus immunohistochemistry on biopsies was negative. His UC remained refractory despite accelerated infliximab (3 doses of 5 mg/kg) and vedolizumab (2 doses of 300 mg) over 3 weeks with persistent bloody diarrhea of 6-8 bowel actions per day, CRP 40 mg/L, and albumin 22 g/L.

A decision was made to perform a rescue diverting loop ileostomy instead of a colectomy; concurrently, a left hemi-hepatectomy for a 58-mm segment 4a metastatic melanoma deposit was performed. His disease activity was later controlled with 8-weekly vedolizumab, reaching histologic remission. His ileostomy was reversed 14 months later. He achieved clinical remission with a return to normal functional status 2 months later. Colonoscopy 1 year following ileostomy reversal showed his UC to be in histologic remission on maintenance of 8-weekly vedolizumab. His melanoma remains in remission 3 years after the last dose of immunotherapy.

Infliximab and vedolizumab are commonly used for steroid-­refractory checkpoint-inhibitor colitis, with or without underlying IBD.^2^ However, 10%–30% of patients fail to respond, potentially requiring colectomy.^2^ Diverting loop ileostomy is not routinely recommended for patients with refractory UC, with ultimate successful ileostomy reversal in a small minority.^3^ Diverting loop ileostomy in checkpoint-inhibitor colitis has been described, but reversal may be unsuccessful due to recurrence of severe colitis.^4^ As this case shows, diverting loop ileostomy may be an alternative to emergent colectomy when worsening of UC is associated with severe checkpoint inhibitors. This may enable medical optimization and time for checkpoint-inhibitor washout prior to ileostomy reversal.

## Data Availability

No new data was generated in support of this research.
